# Persistence of Lineage IV Peste-des-petits ruminants virus within Israel since 1993: An evolutionary perspective

**DOI:** 10.1371/journal.pone.0177028

**Published:** 2017-05-18

**Authors:** Brian Clarke, Mana Mahapatra, Orly Friedgut, Velizar Bumbarov, Satya Parida

**Affiliations:** 1 The Pirbright Institute, Surrey, United Kingdom; 2 Kimron Veterinary Institute, Rishon LeTzion, Israel; University of Minnesota, UNITED STATES

## Abstract

Peste-des-petits ruminants (PPR) is one of the most important infectious diseases of domesticated small ruminants. From the initial identification in 1942 in West Africa, PPR virus (PPRV) has spread throughout much of the developing world. PPRV is now considered endemic throughout Africa, with the notable exception of South Africa, the Middle-East and Israel, as well as South-, East-, and Central Asia. Despite this widespread dispersal, the evolution and transmission of PPRV in endemic populations is not well understood. This understanding will be critical in the planning of rational measures to eradicate PPRV by the planned time as defined by the FAO and OIE. To further advance the understanding of the evolution of PPRV the full genome sequence of 18 viruses isolated from Israel from consecutive years between 1997–2014 were generated. This data set is unique and crucial for the understanding of the evolution of PPRV, as it represents the first set of full-length sequence data available from consecutive years from a single geographic location. Analysis of these full genome sequences shows 96.2–99.9% nucleotide conservation across the Israel isolates and further demonstrates the strong purifying selection pressures on PPRV within Israel and globally. Four amino acid substitutions indicative of putative positive selection were additionally identified within the Israel isolates. The mean substitution rate per site per year was estimated to be 9.22 x 10^−4^ (95% HPD 6.206 x 10−4–1.26 x 10^−3^). Using Bayesian and phylogenetic analyses we further demonstrate that the PPRV isolates from Israel belongs to linage IV and form a single strong regional cluster within all other lineage IV viruses circulating worldwide implying a single incursion into Israel.

## Introduction

Peste-des-petits ruminants (PPR) is an economically significant and highly contagious, OiE listed disease of small ruminants. PPR virus (PPRV) is considered endemic throughout Africa with the current exception of South Africa, the Middle East, and Asia. The etiological agent PPRV is a small (15,948 nt) single-stranded negative-sense RNA virus in the genus *Morbillivirus* and the family *Paramyxoviridae*. PPR was first identified as a separate disease from the closely related Rinderpest in 1942 in Côte d'Ivoire [1, 2]. Although small domestic ruminants are the principal host, PPRV has been reported to infect either clinically or sub-clinically several other species, including cattle [3, 4], camelids (camels and dromedary) [5], buffalo[6, 7], gazelle [7], Asiatic lion [8] and other wild ungulates [9, 10]. As PPR is found almost exclusively in the developing world [11, 12], PPRV infection of sheep and goats in these vulnerable communities has devastating results on the economy and food security of these communities. Following the formal declaration of the eradication of rinderpest in 2011, the sister disease PPR was determined to be a suitable target for eradication. Despite declarations of a 2030 target for PPR eradication [12, 13], restricted vaccination strategies employed thus far in addition to PPR being endemic in some of the poorest countries globally and the associated porous borders of PPRV endemic countries [14] has resulted in the rapid spread of PPR [11, 15].

The molecular epidemiology of PPRV, which is primarily based on sequence comparison of a small variable region of the C-terminus of the nucleoprotein (N) gene (1360–1614, 255 nt long) as well as partial fusion protein (F) gene (256–575,319 nt long) [16], has identified 4 distinct lineages (I–IV) [17, 18]. PPR was first reported in the Middle East in Oman in 1978 [19] and the UAE in 1986 [20], full genome sequencing of these viruses has determined these outbreaks to be caused by lineage III PPRV [17]. The next confirmed report of PPRV within or around the Middle East was in Israel in 1993 [21]. Phylogenic analysis of partial N gene of PPRV revealed this virus to be from lineage IV, unlike the Oman and UAE viruses. Subsequently, lineage IV PPRV has been reported throughout the Middle East with the exception of Yemen [22, 23] and Oman [19] where only lineage III has been identified. Lineage IV has a wide geographic range from China and Tibet [24] to the Middle East [25, 26], and has most recently expanded into Northern [27, 28], Central [29], and Eastern Africa as far south as Tanzania [6, 7]. Although why lineage IV is more widespread than other lineages is unknown, it is possible that lineage IV may have a selective advantage over other lineages. As with lineage IV, Lineage III is present in both Eastern Africa [30] as well as the Middle East [31]. Lineage I remains confined to Western Africa [32], and lineage II has recently spread from Western Africa to Central and Eastern Africa [7]. At the time of this manuscript preparation (12/01/2017) 14 Nubian Ibex (Capra nubiana) contained within a large enclosure at the great biblical zoo at Jerusalem have been reported to be infected with PPRV (awaiting lineage confirmation) further emphasizing the current topical importance of PPRV within Israel [33].

Sporadic sequencing in response to outbreaks and poor availability and annotation of samples and sequence data has hampered investigation into the evolution and spread of PPRV. Many aspects of PPRV evolution, such as ancestral virus location, divergence and time of origin, and historical and geographic patterns of spread are poorly understood. A better understanding of the evolution of PPRV would enable prediction of how these viruses will lead to further outbreaks and epidemics, and provide data for control strategies. To understand the genetic and evolutionary influences on the spread of PPRV a substantial increase of PPRV sequence data is required which can provide insight into sites under significant positive selection. In this study phylogenetic and evolutionary analysis of 55 full length genomes including 18 viruses from Israel collected from outbreaks between 1997 and 2014 alongside 192 variable c-terminal N regions provides insight into the evolution and spread of PPRV within Israel as well as globally.

## Materials and methods

### Sample collection

The samples were collected as part of routine diagnostic work performed by the Israel veterinary services and submitted to the Kimron Veterinary Institute, Israel for diagnosis. Samples consisted primarily of lung tissues collected from suspected animals during post-mortem and nasal/ocular swabs from suspected diseased animals. Selected samples from the confirmed cases were shipped in dry ice to the Pirbright Institute, UK for molecular analysis. The details of the Israel derived samples are presented in Table 1.

**Table 1:** Sample details.

Sample Number/ year	Animal	Sample Type	Location	Clinical symptoms	PCR
2536/97	sheep	Lung Tissue	Hebron	Diarrhoea, anorexia, nasal & ocular discharge	+
4522/98	goat	Lung Tissue	Tzora	death after 6 days of PPR-like signs	+
5236/00	sheep	Lung Tissue	Ofer	cough, nasal discharge, conjunctivitis	+
5921/01	sheep	Lung Tissue	Nazareth	pneumoenteritis, hemorrhagic colon	+
7161/01	goats	Lung Tissue	Kseifa	pneumonia, death	+
6586/01	sheep	Lung Tissue	Zarzir	diarrhoea, rhinitis, conjunctivitis, fever, gingivitis	+
2105/03	sheep	Lung Tissue	Zarzir	diarrhoea, stomatitis, pneumonia, fever	+
2233/03	sheep	Swabs	Beir El Makhsour	stomatitis, pneumonia, enteritis	+
1012/04	goat	Lung Tissue	Tel Arad	diarrhoea, death	+
1277/04	sheep	Lung Tissue	Jordan Valley (Oudja)	cough, mouth lesions, constipation, lameness	+
1031/05	sheep	Lung Tissue	Deir el Assad	fever, nasal/ocular discharge, stomatits,	+
1034/05	sheep	Lung Tissue	Deir el Assad	fever 41.5, nasal/ocular discharge, stomatits,	+
1251/05	sheep	Lung Tissue	Netua	pneumonia, diarrhoea, death	+
1483/08	goat	Lung Tissue	Atauna	conjunctivitis, stomatitis, pneumo-enteritis	+
1929/11	goats	Lung Tissue	Goren	classic PPR	+
1947/11	goat	Lung Tissue	Goren	classic PPR	+
1192/12	sheep	Lung Tissue	Rahat	pyrexia, conjunctivitis, diarrhoea	+
1571/14	sheep	Lung Tissue	Um El Fahem	respiratory distress, salivation, rhinitis	+

### Ethical statement

Samples were collected for the diagnosis of the disease under the usual veterinary service work in Israel, no permits were required for collection. The samples were sent to the Pirbright Institute (holds the PPR reference laboratory) for further diagnosis and molecular characterization. When consulted, local Pirbright animal welfare ethical review board (AWERB) confirmed that no requirement of approval needed as the samples were collected primarily for veterinary diagnosis purpose at Israel and not for the research. Further the tissue samples were collected form dead animals only.

### RNA extraction and Reverse Transcription—Polymerase Chain Reaction (RT-PCR)

Full length genome sequencing was performed on tissue samples using a hemi-nested RT-PCR as described previously [34]. Briefly, approximately 0.3 gm tissue pieces were ground in M25 buffer using a mortar and pestle. The homogenate was clarified by centrifugation at 3,000 rpm for 15 minutes at +4°C followed by filtration through a 0.22 um filter. 500 μl tissue extract or 100μl swab fluid was added to 1 mL TrizolTM and the manufacturers directions followed. Initial first-strand synthesis and first round PCR was performed as described in the above paragraph except first-strand synthesis was performed at 48°C and an annealing temperature of 55°C was used. Second round amplification was accomplished using the KOD hot-start polymerase kit and amplification of the terminal 5’ and 3’ ends of the PPRV genome was accomplished by Random Amplification of cDNA Ends (RACE), as previously described [34, 35]. The PCR amplicons were purified using the GE Healthcare Illustra GFXPCR purification kit (GE Healthcare, Pittsburgh, USA) according to the manufacturer’s instructions and sequenced using BigDye^®^ Terminator v3.1 Cycle Sequencing Kit (Applied Biosystems, Carlsbad, USA) on an ABI 3730 machine. Sequences were assembled and analyzed using SeqMan pro (DNAStar Lasergene 13.0). Nucleotide sequences of the viruses were aligned using the CLUSTAL X multiple sequence alignment program [36] or MUSCLE [37] as appropriate.

### Sequence datasets

37 complete PPRV genome sequences (S1 Table) were retrieved from GenBank on 29/11/2016. In addition representative partial N sequences (n = 73) (S2 Table) from around the world were included in the analysis. Sequences obtained from live attenuated vaccine strains (Sungri 1996: KJ867542, KF727981) and Nigeria 1975 (X74443, HQ197753) were removed prior to analysis as these sequences have previously been shown to substantially skew phylogenetic analyses [32]. Additionally the 18 Israeli sequences generated in this study were included in the analysis either as full length genomic sequences or the partial N sequence making a total of 55 full genome and 91 partial N-gene sequences.

### Model selection and phylogenetic analyses

In total eight models were tested to select a statistically appropriate model to study the evolution of PPPRV using the full-length genome dataset (S1 Table). The tested models included combinations of the Hasegawa-Kishino-Yano (HKY) and the general time-reversible (GTR) substitution models, with inclusion or exclusion of a gamma distribution among-site rate heterogeneity, and strict or uncorrelated lognormal relaxed molecular clock (HKY Gamma strict, HKY strict, HKY Gamma relaxed, HKY relaxed, GTR gamma strict, GTR strict, GTR Gamma relaxed, & GTR relaxed) [38]. For each model, parameters were estimated using the stepwise Bayesian Markov chain Monte Carlo (MCMC) approach implemented in BEAST 2 [39]. MCMC chains were run for 100,000,000 states and sampled every 10,000 states. MCMC convergence was evaluated using Tracer 1.7 (http://beast.bio.ed.ac.uk). The models were compared using Bayes Factors (BF), to test the difference in the marginal log likelihood between the models (40). To identify the most recent common ancestor and the likely dates of divergence, the 18 Israel sequences were compared to all available full-length PPRV genomes (n = 37), using a coalescent-based Bayesian MCMC [40, 41] approach. The partial N dataset (n = 81) was aligned using MUSCLE and phylogenetic analyses were performed using MEGA6 [42]. The neighbour-joining tree was generated using the Kimura 2-parameter model and tests for phylogeny performed using the bootstrap method with 20,000 replications and the gaps/missing data removed by pairwise deletion [43].

### Tests for positive selection

Utilizing multiple site-specific codon substitution models, the protein-coding regions of N, P, M, F, H and L genes of PPRV were tested to determine the sites under likely positive selection. The respective protein-coding regions of all 55 viruses were extracted from their respective full genome sequences and used for homologous recombination analyses. Before any estimation of selection was performed a pair of recombination detection programs; Search for Break Point (SBP) and Genetic Algorithm Recombination Detection (GARD) were employed to determine if any of the genes showed evidence of recombination [44]. Both these analysis tools are implemented as part of the Datamonkey platform (www.datamonkey.com) [45].

Maximum-likelihood based selection analysis was performed to determine whether any of the genes are evolving under positive selection. In order to compare the evolution of PPRV across lineages, as well as within lineage IV, and within Israel the available full length genomic sequences were divided into three groups, firstly all available sequences from all lineages (n = 37), all available Lineage IV sequences (n = 22), and the Israel sequences from this study (n = 18). Several codon-specific models: Single-Likelihood Ancestor Counting (SLAC) and Random-Effect Likelyhood (REL) were employed to test the rate of nonsynonymous (dN) to the synonymous (dS) ratio (dN/dS) which vary among residues [46]. Positively selected sites were also detected using the mixed effects model of evolution (MEME) method to infer episodic selection [47]. Selection analyses were performed independently using the three groups of data as described above. P values >0.1 and Bayes factors greater than 50 were taken as significant following SLAC, MEME and REL analyses, respectively as per the defaults set for the Datamonkey site. Sites identified as significant by at least two models were considered positive.

## Results

### Sample location and sequence homology

**Fig 1:**
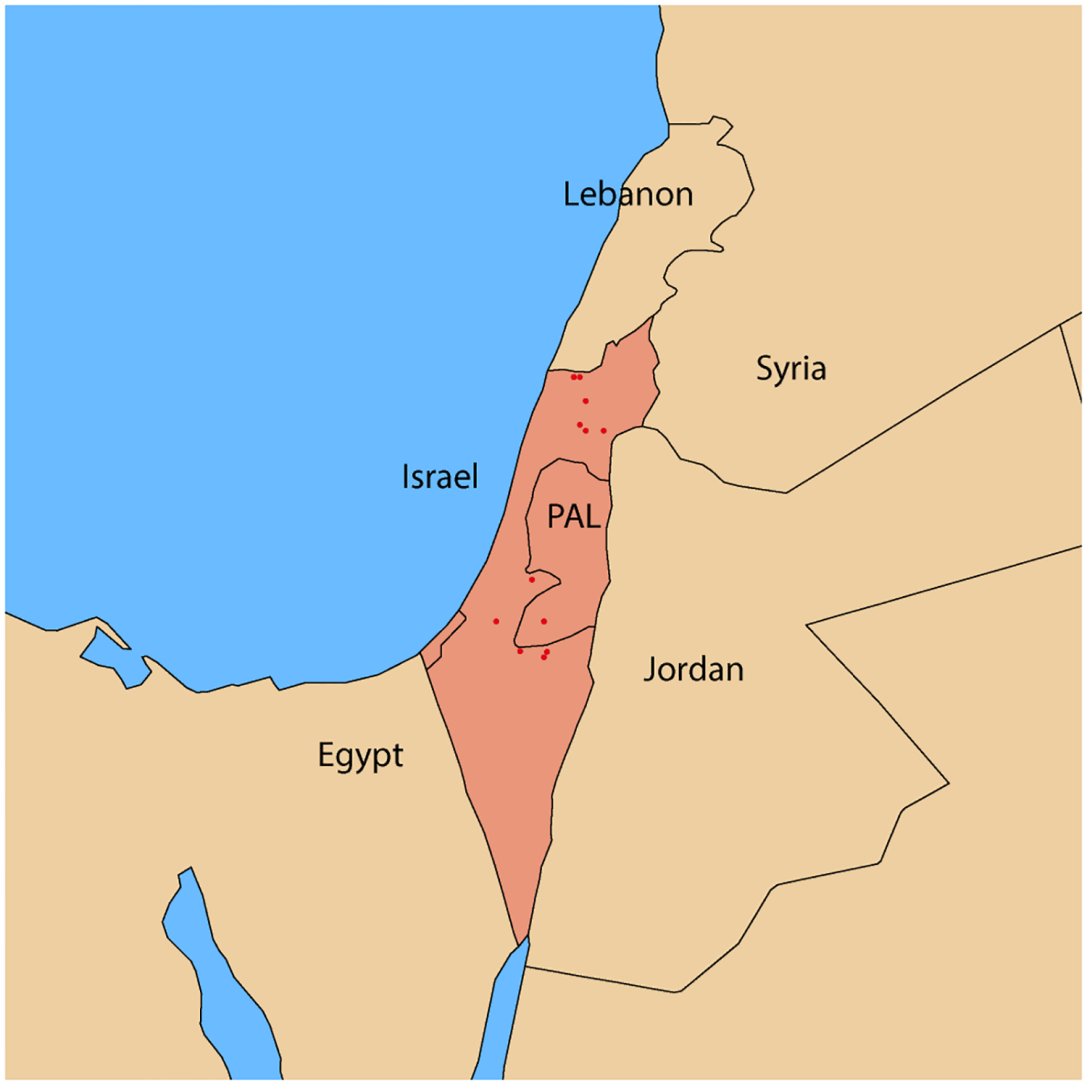
Location of PPR outbreaks in Israel (1993–2014). Sampling locations are plotted in red, GPS co-ordinates derived from Google maps API [49]. Comparisons of the full length genome, and coding regions from the 18 Israel samples sequenced in this study. Indicated that the Israel isolates were more closely related than other groups (Table 2). Overall the Israel sequences were found to be 96.2 to 99.9% identical at the nucleotide level. As has been previously reported the P gene was the most variable [50] and the matrix (M) gene the most conserved (Table 2).

Eighteen PPRV full genome sequences were generated from PPR confirmed tissue samples and nasal swabs from Israel collected during 1997–2014 (Table 1). All viruses sequenced in this study were confirmed as lineage IV and are of 15948 nt long conforming to the rule of six as previously reported for all morbilliviruses [48]. History of outbreaks are presented in Table 1 and sampling locations are plotted in Fig 1.

**Table 2:** Nucleotide and amino acid percentage differences between PPRV Lineages.

	Full Length	N		P		M		F		H		L	
		NT%	AA%	NT%	AA%	NT%	AA%	NT%	AA%	NT%	AA%	NT%	AA%
All available Lineages (I–IV) (n = 37)	0.01–12.9	0.01–12	0.01–8.4	0.01–15.0	0.01–8.6	0.01–11.2	0.01–5.1	0.01–11.6	0.01–6.1	0.01–12.3	0.01–12.3	0.01–10.8	0.01–5.5
Available Lineage IV (n = 22) (1996–2016)	0.01–5.1	0.01–5.5	0.01–5.2	0.01–14.5	0.01–8.8	0.01–4.9	0.01–3.9	0.01–8.3	0.01–3	0.01–4.9	0.01–9.2	0.01–6.8	0.01–2.4
Israel (n = 18) (1997–2014)	0.01–3.83	0.01–4.13	0.01–3.9	0.01–8.3	0.01–7.5	0.01–3.7	0.01–2.9	0.01–6.22	0.01–2.3	0.01–3.7	0.01–6.9	0.01–5.1	0.01–3.8

### Measuring selection pressures

Two independent assessments of homologous recombination were performed on the aligned coding regions of all genes from the three full length genomic data sets to ensure homologous recombination was unlikely to be a significant factor in the evolutionary pressures acting on PPRV. A single putative homologous recombination event was observed following GARD analysis however it was poorly supported with a relatively high cAIC value (71.7657) as such this is unlikely to be a significant factor in the evolution of PPRV.

**Fig 2:**
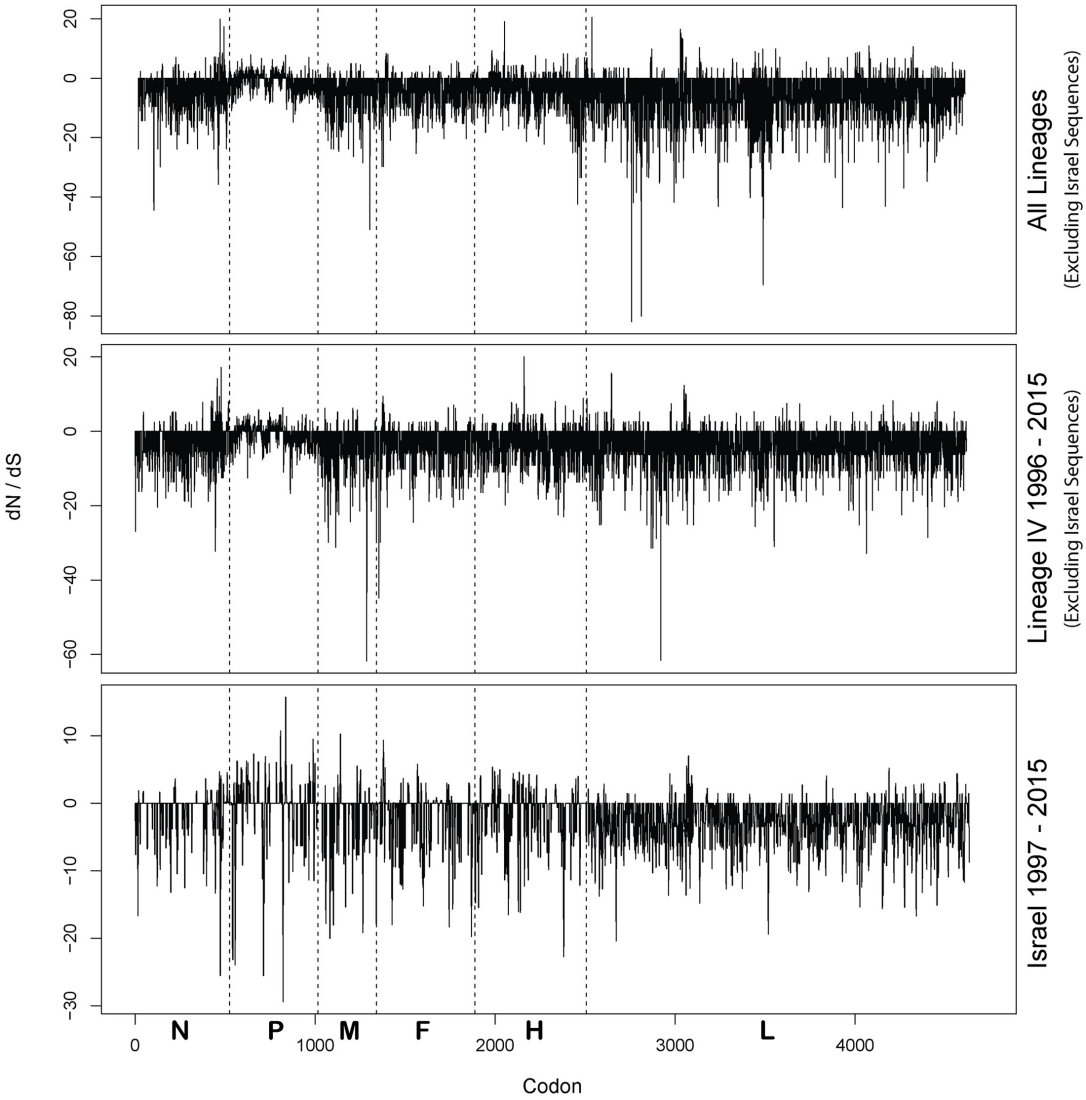
Mean ratio of nonsynonymous (dN) to synonymous (dS) substitutions for each site in the coding regions of PPRV. Proportions of dN and dS were calculated using the single-likely ancestor counting method [45]. Dashed lines indicate gene boundaries.

To determine the degree of selection acting upon the PPRV genomes three different tools were used. The coding regions of each gene of three groups were analyzed separately, all available non vaccine full length genomes (Lineage I-IV), all available non vaccine Linage IV genomes and all full length lineage IV Israel sequences generated in this study. The dN/dS values per site as determined by SLAC are shown in Fig 2 as an example and indicate that the PPRV genome is primarily under negative selection. The overall dN/dS values of coding region are presented in Table 3 for each group of samples. All genes for all groups showed dN/dS values >1 for all analyses further demonstrating the strength of purifying selection applied across the breadth of the PPRV genome across all groups.

**Table 3:** Mean dN/dS values for PPRV coding regions following analysis for positive selection.

	N		P		M		F		H		L	
	SLAC	REL	SLAC	REL	SLAC	REL	SLAC	REL	SLAC	REL	SLAC	REL
All available Lineages	0.13892	-0.86	0.47	-0.53	0.07	-0.93	0.11	-0.89	0.44	-0.8	0.1	-0.9
All available Lineage IV (1996–2016)	0.146	-0.85	0.47	-0.53	0.07	-0.93	0.1	-0.8	0.19	-0.8	0.09	-0.91
Israel (1997–2014)	0.197	-0.8	0.64	-0.41	0.18	-0.82	0.15	-0.86	0.3	0.349	0.1	-0.91

A number of sites were identified as being under positive selection (Table 4). To minimize the likelihood of sites being incorrectly identified sites were only considered as significant if they were identified by at least two of the three selected analysis methods. Interestingly residue 421 in the polymerase (L) gene was identified in all groups of viruses as a leucine (L) to asparagine (N) shift. Additionally, there were a number of changes observed in the hemagglutinin gene, particularly codon 246 which exhibited multiple amino acid substitutions (L-I,L-P,P-S,L-P,P-I) as described in Table 4. These changes occurred in multiple branches in both the Lineage I–IV groups as well as the Lineage IV group. Changes included reversion back to the original leucine from proline and conservative changes from both proline and leucine to isoleucine. This amino acid change was not observed in the Israel group, amino acid 246 of the hemagglutinin gene remained as a fixed leucine.

**Table 4:** Significant amino acid changes identified in PPRV lineage groups.

	N		P		F		H		L	
	Codon	AA	Codon	AA	Codon	AA	Codon	AA	Codon	AA
All available Lineages (Excluding Israel samples)	456	Pro—Ser	295	Leu—Pro			246	Leu—Pro Pro—Ile Pro—Leu Pro—Ser	421	Ile—Asp
	478	Leu—Ser Ser—Pro Ser—Leu								
All available Lineage IV (1996–2016) (Excluding Israel samples)			217	Asp—Met	8	Thr—Val Ile—Val	223	Val—Fle	68	Val—Leu
			261	Glu—Lys			246	Leu—Pro Pro—Leu Pro—Ile Pro—Ser	421	Ile—Asp
			388	Val—Ile			309	Ser—Gly	1120	Lys—Gln
									1696	Thr—Ile
									2097	Leu—His
									2162	Thr—Ala
Israel (1997–2014)			466	Tyr—Ser	201	Lys—Val			421	Ile—Asp
									721	His—Pro His—His

### Estimating PPRV Divergence

To estimate the time to most recent common ancestor and mean substitution rates for PPRV lineages we performed a number of Bayesian analyses using all available full length PPRV genomes excluding vaccine viruses (n = 37) and those sequenced in this study (n = 18), and the coding regions of the same groups. The best-fitting model was GTR with a relaxed exponential molecular clock (UCED) (2lnBF < 30), and was thus used in subsequent analyses. This model has been previously used to estimate PPRV evolutionary rates [32, 51], as there was no significant difference between the UCED clock models (2lnBF < 1) the exponential model was selected as it provided the lowest 95% HPD intervals. The mean substitution rate per site per year was estimated to be 9.22 x 10^−4^ (95% HPD 6.206 x 10−4–1.26 x 10^−3^) and was consistent across coding regions.

**Fig 3:**
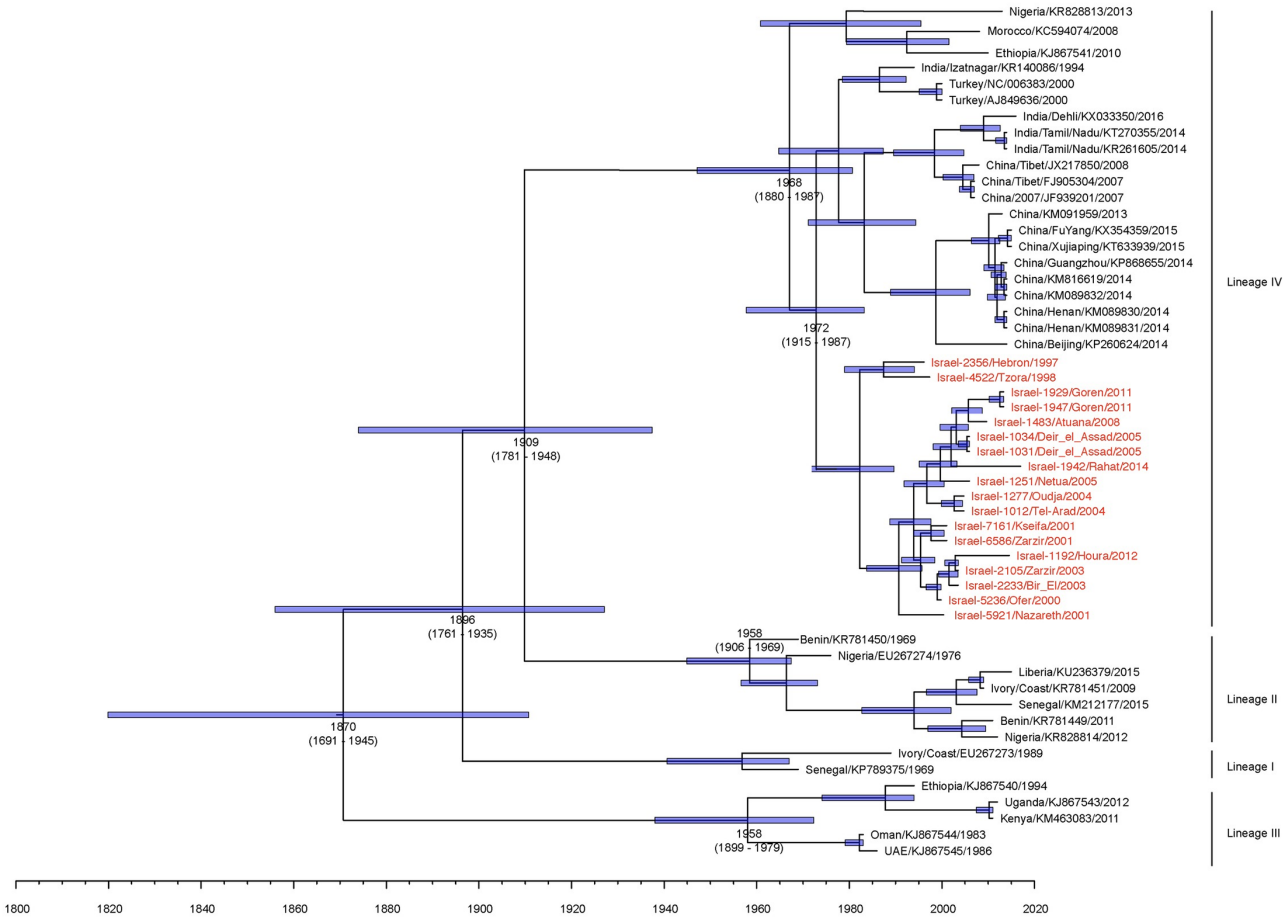
Maximum clade credibility (MCC) tree from Bayesian analysis of full-length PPRV genomes. The posterior probabilities are indicated by the size of the node, and TMRCA and 95% HPD of the branches are depicted. Accession number, country of origin, and sampling year of each isolate is shown. All sequences generated in this study are highlighted in red.

To visualise the analysis a Bayesian MCC tree was constructed based on the maximum sum of the posterior probabilities from the full-length genome sequences [41] (Fig 3). Lineage III was the first lineage to diverge and was estimated to have emerged in 1870 (95% HPD 1691–1945). Lineage I was estimated to have diverged in 1896 (95% HPD 1761–1935), Lineage II in 1909 (95% HPD 1781–1948) and Lineage IV in 1958 (95% HPD 1880–1987). Interestingly, all the full-length genome sequences from Israel strongly grouped together (Fig 3). Within the Israel virus sequences there are two obvious sub-clades both of which were well supported by Bayesian prior and bootstrap values.

**Fig 4:**
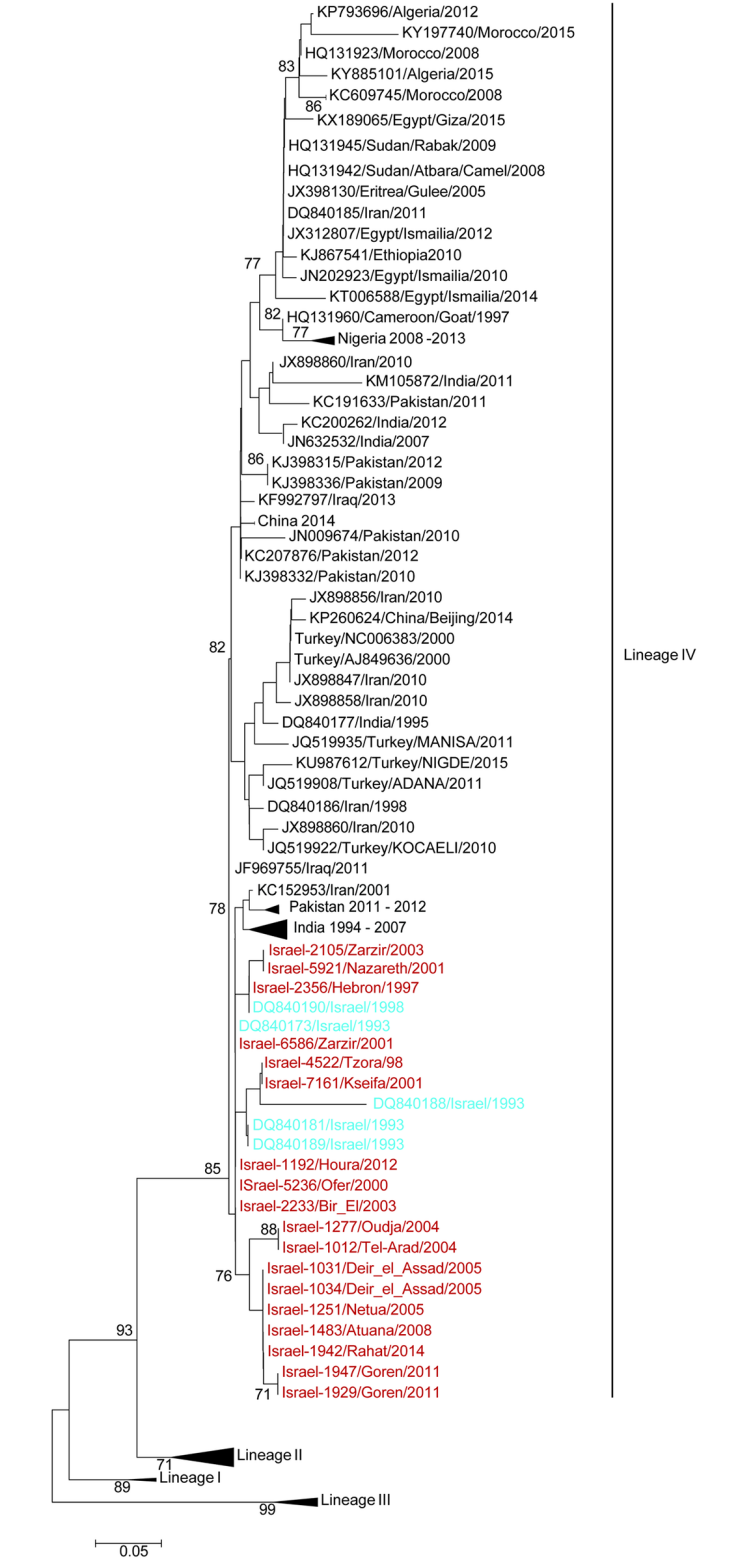
Neighborhood-Joining tree of all unique partial N PPRV Isolates. Accession number, country of origin, and sampling year of each isolate is shown. Significant bootstrap values are included at the nodes. All sequences generated in this study are highlighted in red and isolates previously sequenced from Israel are highlighted in blue.

As there are no other Middle East lineage IV viruses for which full length genome sequences are available a neighbourhood joining tree was constructed using the C-terminal partial N region using available sequences for which an accurate date of collection and location could be obtained (n = 192). Of these 37 are from the Middle East, the majority from Turkey (n = 21) and 6 from Israel as well as 18 from this study (Fig 4). As was observed with the full length genomic sequences the Israel isolates form a unique strong clade even with the substantially increased dataset. As with the temporal analysis the Israel isolates sequenced in this study and the 6 partial N isolates previously sequenced from Israel between 1993–1998 form a clear single clade with no mixing of viruses from other countries. Most closely related to this clade of Israel derived viruses are isolates from Iran (KC15953 2001) and India (isolated from 1994–2005).

## Discussion

PPR was first reported in Israel in 1993, after which Israel has reported consistent outbreaks of PPR to the OIE almost every year. Despite this, there has been to date sparse information available about viruses circulating within Israel only 6 partial N sequences were available from 1993 to 1998. In this study we generated 18 full-length genome sequences of PPRV circulating within Israel between 1997 and 2014. Phylogenetic analysis of the full length genome demonstrates that all these viruses belong to a single unique clade of PPRV lineage IV with very high nucleotide similarity. This suggests that a single initial incursion is likely to be responsible for the persistence of PPRV within Israel. The obvious social, cultural, and political uniqueness of Israel within the Middle East is the likely reason for the apparent lack of transboundary movement of PPRV between Israel and its neighbors. While determining exact routes of animal movement within the Middle East is not easy the FAO estimates around 3,000,000 animals were traded between countries within this region in 2013 of which trade in to and out of Israel accounted for less than .0001% (n = 179) (S3 Table) [52]. This low regional movement of PPRV through Israel and large animal numbers and yearly outbreaks of PPRV makes Israel an unique environment to examine the spread and evolution of PPRV.

There is a lack of available sequence data from the Middle East, with the obvious exception of Turkey. In particular, there are no sequence data available from either Lebanon or Jordan and the clustering of PPRV outbreaks in the northern region bordering Lebanon could suggest some potential incursions from this direction. The historical introduction of PPRV into Israel into the Tiberias district bordering Lebanon [53] as well as the recent reports of the likely transmission of Lumpy Skin disease from Lebanon into Israel [54, 55] is substantial evidence that there is a potential route of animal migration in this region. As has been previously discussed multiple independent clades of a virus within a country is potentially indicative of multiple incursions or indeed possibly viral evolutionarily holdups or bottlenecks [51]. That there are no inclusions of other viruses within the Israel population suggests that a single incursion of PPRV into Israel has potentially occurred. However as there is a dearth of sequence data available from the surrounding countries due to a variety of political and cultural reasons, the presence of missing sequences that may indicate multiple incursions cannot be discounted.

Within Israel and the Palestinian Autonomous Territories (Gaza and the West Bank) (PAT) there are a wide range of farming methods of sheep and goats which can be categorized as intensive, semi-intensive and extensive. The official estimate suggests there are approximately 435,000 sheep and 90,000 goats (2015) in Israel [56]. Unfortunately very little information is available about farming practices in PAT. However, the most recent livestock survey in 2013 suggests that there were 730,894 sheep and 215,335 goats; approximately double the number present in Israel at the same time period, the overwhelming majority (96.2% and 95% respectively) located in the West Bank [57]. However, despite the large number of animals present in Palestine relative to Israel only two of the samples in this study were from PAT controlled areas, Hebron and the Jordan Valley (Oudja). The obvious interconnection between these samples and samples derived from the same year (2004) within Israel between Tel Arad and Oudja (Uja, Al-Auja) confirms anecdotal accounts of animal movement between the PAT and Israel [58]. The current security fences around the PAT to separate it from Israel began to be raised in 2005, despite this, there are anecdotal reports of animal movement from the southern West Bank to the Negev [58]. This uncontrolled movement of animals from the PAT may be responsible for the repeated PPRV outbreaks in Israel despite mass vaccination strategies being employed.

Following the initial incursion of PPRV into Israel in 1993 prior to being discontinued in June 2016, vaccination of small ruminants was performed annually and young animals were vaccinated for the first time at 3–4 months of age. Despite this practice PPR outbreaks have been observed consistently as reported in this publication. The occurrence of repeated outbreaks of PPR despite a mass vaccination strategy suggests reintroduction of unvaccinated animals—either through non-compliance of vaccination, or regular introduction of un-vaccinated animals from infected populations such as the West Bank. The latter is potentially suggested by the sequence analyses performed in this study that indicates strong connections between Tel Arad and the Jordan Valley in 2004.

As with most negative-sense single stranded RNA viruses, homologous recombination does not appear to play a major role in the evolution of PPRV. Further, consistent with other paramyxoviruses the PPRV genome is consistently under negative/stabilizing selection across the whole genome and all genes and coding regions. Indeed, our data suggests that PPRV has persisted within Israel for more than 20 years with less than 4% nucleotide changes across the 18 viruses sequenced. This conservation of PPRV as a single conserved clade within a geographic region is in contrast with viruses isolated from the Indian sub-continent and Africa, where significant intermingling can be observed [59].

Unlike previous studies of available full length genomic sequences [32] or of lineage IV sequences [51] we identified a small number of sites potentially under significant positive selection. This could be due to the level of purifying selection much of the genome is under. Purifying selection of RNA viruses has repeatedly been demonstrated to mask diversifying selection processes and PPRV conforms to this expectation [60]. Multiple methods including mixed effect and branch-weighted models can substantially increase the likelihood of identifying sites truly under positive selection, however care must be taken to minimize type 1 errors by selecting sites identified by multiple models. Two sites identified as being under positive selection are of particular interest in the evolution of PPRV codon 246 in the H gene and 421 in the L gene. Interestingly H 246 showed repeated reversions to its basal residue (reverting from leucine to proline) multiple times as well as shifting to the conservative isoleucine. Frequent reversals such as this have typically been shown to indicate fluctuations in the immune status of populations as the levels of herd immunity shift [61]. As such, this frequently reverting site may be representative of the *ad hoc* nature of PPRV vaccination to date or variance between host groups.

Estimations of evolutionary rates by molecular clock analyses of closely related species such as virus groups are important tools to estimate the rate of mutation and likely sources of outbreaks, and common ancestors between virus groups. The molecular clock rates estimated by our analysis are consistent across groups which is consistent with previous estimates of substitution rates of PPRV (1.64 x 10^3^–9.13 x 10^4^ substitutions per site per year [11, 32, 51]) as well as other paramyxoviruses (103–10^4^ [51, 62]). The TMRCA of all PPRV lineages was estimated to be 1870 (95% HPD 1691–1945), this is somewhat earlier than previous estimates using fewer virus sequences as could be expected from the greater geographic and temporal scale represented in this sample set. This TMRCA estimation is likely to be substantially distorted by the substantial gaps of available sequences particularly prior to the 1990’s. The first identification of PPRV as distinct from its sister virus rinderpest occurred in 1942. PPRV and RPV exhibit substantial cross neutralization and similarity in clinical signs, and this combined with the lack of molecular tools for diagnostics and differentiation account for the delays between the putative TMRCA and the first identification of PPRV. An additional factor clouding the TMRCA dates of ancient PPRV sequences is the strength of negative selection which the PPRV genome is under in addition to the overwhelming prevalence of recent samples <1990 which may additionally substantially bias the calculated TMRCA dates [32].

Determination of exact routes of transmission of PPRV is not easy as there are many outbreaks for which there is no historical sequence data, in particular isolates from outbreaks in the 1960–80s for which there is only serological data available. As such, the exact origin of PPRV Lineage IV as well as the route of PPRVs eastward transmission from Africa into the Middle East and Asia is currently lost to time. However further sequence analysis of PPRV Lineages in particular Lineage IV may help to identify any virological factors that may have facilitated the grand spread of PPRV lineage IV in comparison to the other lineages.

## Conclusions

Despite the widespread regional and endemic nature of PPRV infection our phylogenic analysis of the full genome PPRV Israel sequences suggest that PPRV has persisted consistently within Israel, with limited intermingling with other regions following the initial incursion. Uniquely amongst all of the equivalent groups of isolates the Israeli samples cluster together as a single strong clade with no outlying sequences or regional intermingling, such definitive regional clustering is not apparent for any other region.

The identification of persistent PPRV infections within Israel following the original incursion around 1993 with apparently no significant introductions since this time has significant implications for the eradication of PPRV within Israel. As was observed in Morocco following the original 2008 outbreak systematic mass vaccination is an effective method of eradication of PPRV in a geographic region [27]. The importance of establishment of a reliable and effective vaccination strategy for PPR in Israel and the PAT is further emphasized by the identification of infected Nubian Ibex within the Great Biblical Zoo of Jerusalem in January 2017. These animals are reported to have not come in contact with any newly imported animals nor domestic ruminants suggesting the potential route of infection via humans or fomites [33]. Due to its unique geopolitical environment and border controls Israel has an opportunity to employ an effective mass vaccination strategy jointly in Israel and PAT to eradicate PPR from Israel and the Palestinian Autonomous Territories.

## Supporting information

S1 Table Partial N sequences utilized in the preparation of Fig 4 retrieved from NCBI and annotated via google maps 29/11/2016. (XLSX)

S2 Table Full-length PPRV Sequences utilized in this study retrieved 29/11/2016 from NCBI. (XLSX)

S3 Table Animal movement numbers for sheep and goats extracted from the FAO. (CSV)
